# The concept of “Four Walls, Two Poles” in the lesions of the thalamus and ganglion regions: case report and literature review

**DOI:** 10.1186/s12893-021-01059-9

**Published:** 2021-01-22

**Authors:** Haiyang Yang, Gang Bai, Yongli Zhang, Guolong Chen, Lei Duan, Youyun Bi, Haifeng Yang

**Affiliations:** 1 Department of Neurosurgery, Kunming Sanbo Brain Hospital, Kunming, 650010 China; 2 Department of Neurosurgery, Yunnan Cancer Hospital, Kunming, 650118 China; 3 Department of Neurosurgery, Sanbo Brain Hospital, Capital Medical University, Beijing, 100069 China

**Keywords:** Thalamic glioma, Microsurgical techniques, Four walls two poles

## Abstract

**Background:**

There are few articles about the surgical techniques of thalamic glioma and the lesions in the basal ganglia area. According to three existing cases and the literature review (Twelve articles were summarized which mainly described the surgical techniques), we discuss the surgical characteristics of lesions of the thalamus and basal ganglia area and summarize the relevant surgical skills.

**Case presentation:**

Of the three cases, two were thalamic gliomas and one was brain abscess in basal ganglia. According to the three-dimensional concept of the “Four Walls, Two Poles”, lesions of the thalamus and basal ganglia were surgically removed, and the operative effect was analysed by relevant surgical techniques. Surgical resection of the lesions of the thalamus and basal ganglia area according to the three-dimensional concept of the “Four Walls, Two Poles” has achieved good surgical results. Relevant surgical techniques, such as the use of retractors, the use of aspirators, the choice of surgical approaches, and the haemostasis strategy, also played an important role in the operation process.

**Conclusions:**

In the presented three cases the three-dimensional concept of the “Four Walls, Two Poles” allowed for safe surgical resection of lesions of the thalamus and basal ganglia.

## Background

The surgical treatment of thalamic tumours and lesions in the basal ganglion area have been under debate due to their deep localization and adjacent critical structures. Recently, with the continuous development of neurosurgical technology, this area is no longer a restricted surgical area. The present study demonstrated that patients with thalamic high-grade glioma without invasion into the pyramidal tract and brainstem can be considered candidates for surgical resection [1]. According to the origin of the lesions and the direction of growth, an appropriate surgical approach can be used to safely remove the lesions. Surgical techniques play a decisive role in the operation of lesions in this area. In the previous literature, there are few reports about thalamic glioma and lesions in the basal ganglia. The description of surgical resection mainly focused on the choice of surgical approach. There is even less description of the surgical technique. In this paper, we presented a classification of the morphology of thalamic glioma and lesions in the basal ganglia and described in detail the surgical resection strategies and techniques. The application of the concept of "Four Walls, Two Poles" ("Four Walls" refers to the dorsal wall, the ventral wall, the lateral wall and the medial wall. "Two Poles" refers to the anterior pole and the posterior pole.) in the operation of the thalamus and basal ganglia was discussed in the context of three cases.

A PubMed search of published studies of patients who underwent surgery for thalamic gliomas was performed on March 17, 2020. “Thalamic glioma*” and “Surg*” were used as the key words. Only articles written in English and describing patients who underwent surgery were included in this study. Studies that did not describe surgical techniques in detail or that simply performed a stereotactic biopsy were excluded from the final analysis. One of the older articles (written in 1985) was excluded.

The PubMed search yielded 47 articles, of which 35 were excluded based on titles, abstracts and full text screening. The 35 articles did not describe surgical techniques in detail or that simply performed a stereotactic biopsy Finally, 12 articles discussing the surgical techniques, including the present cases, were included in the final review (Table 1).

**Table 1:** Clinical data of the overview of the main findings of the 12 articles of the review

Number	Study	Number of cases	Location of lesions	Treatment planning	Surgical approach	Extent of resection	Survival time
1	Kis et al. [2]	5	3 cases in Rt. Thalamus; 2 cases in Lt. Thalamus	Surgery, RT,and CT	T. cort	STR in 4 cases; PR in 1 case	OS was 2 to 20 months
2	Akiyama et al. [5]	18	3 cases in Rt. thalamus; 2 cases in Lt. thalamus; 13 cases in ventricle, putamen, deep part of frontal lobe	Surgery (under endoscope), RT, and CT	T. cort	GTR in 4 cases; STR in 1 case; PR in 3 cases; biopsy in 10 cases	Without data of follow-up
3	Zhang et al. [4]	33	19 cases in Rt. thalamus; 14 cases in Lt. thalamus	Surgery in 5 cases; surgery and RT. in 16 cases; surgery and CT in 2 cases; surgery, RT and CT in 10 cases	PITI in 5 cases; T. cort in 6 cases; T. vent in 22 cases	GTR in 19 cases; STR in 9 cases; PR in 5 cases	2-year OS rates were 25.9%
4	Kumar et al. [5]	1	Rt. thalamus	Surgery	AIPG	STR	Without data of follow-up
5	Saito et al. [1]	21	6 cases in Rt. thalamus; 15 cases in Lt. thalamus	Surgery, RT, and CT in 21 cases	T. vent; T. cort; transsylvian fissure	GTR in 6 cases; STR in 13 cases; PR in 2 cases	Median OS was 12.6 months
6	Brokinkel et al. [9]	1	Lt. thalamus	Surgery (under endoscope), RT, and CT	T. vent	STR	Without data of follow-up
7	Wu et al. [3]	49	23 cases in Rt. thalamus; 24 cases in Lt. thalamus; 2 cases in Bi. thalamus	Surgery, RT, and CT in 35 cases; Surgery, in 14 cases	T. vent in 42 cases; transsylvian fissure in 7 cases	STR in 20 cases; PR in 24 cases; less than PR in 5 cases	Median OS was 9.0 months
8	Liu et al. [10]	26	13 cases in Rt. Thalamus; 13 cases in Lt. Thalamus	Surgery, RT. and CT, in 23 cases; surgery in 3 cases	T. cort in 18 cases; T. vent in 6 cases; transsylvian fissure in 2 cases	GTR in 5 cases; STR in 15 cases; PR in 6 cases	OS was 2 to 59 months
9	Abdullah Keles et al. 2019	1	Third ventricle	Surgery (under endoscope)	CPeSS	GTR	Without data of follow-up
10	Xiaodong Niu et al. 2020	102	41 cases in Rt. thalamus; 57 cases in Lt. thalamus; 4 cases in Bi. thalamus	Surgery, RT. and CT, in 73 cases; surgery in 29 cases	PITI in 30 cases; T. cort in 8 cases; T. vent in 64 cases	GTR in 46 cases; STR in 50 cases; PR in 6 cases	Median OS was 13.6 months
11	Guo Qinglong et al. 2020	53	Not mentioned in detail	Surgery, RT. and CT, in 42 cases; surgery in 11 cases	TLBG in 35 cases; TCC in 18 cases	GTR and STR in 29 cases; PR in 24 cases	Median OS was 9.0 months
12	Kentaro Chiba 2020	10	2 cases in AT., 3 cases in IC., 5 cases in TP	Surgery, RT. and CT, in 8 cases; surgery in 2 cases	Not mentioned in detail	GTR in 3 cases; STR in 6 cases; PR in 1 case	Median OS was 28.1 months

*Rt* right, *Lt* left, *RT* radiotherapy, *CT* chemotherapy, *T* trans, *Vent* ventricular, *Cort* cortical, *GTR* gross total resection, *STR* subtotal resection *PR* partial resection, *OS* overall survival, *PITP* precentral interhemispheric transcallosal interforniceal, *AIPG* anterior interhemispheric transparaterminal gyrus, *Bi* bilateral, *CPeSS* contralateral perimedian supracerebellar suprapineal, *TLBG* translateral brain gyrus approach, *TCC* transcorpus callosal approach, *AT* anterior type, *IC* thalamointernal- capsular, *TP* thalamopulvinar type

## Case presentation

### Case 1

**Fig. 1:**
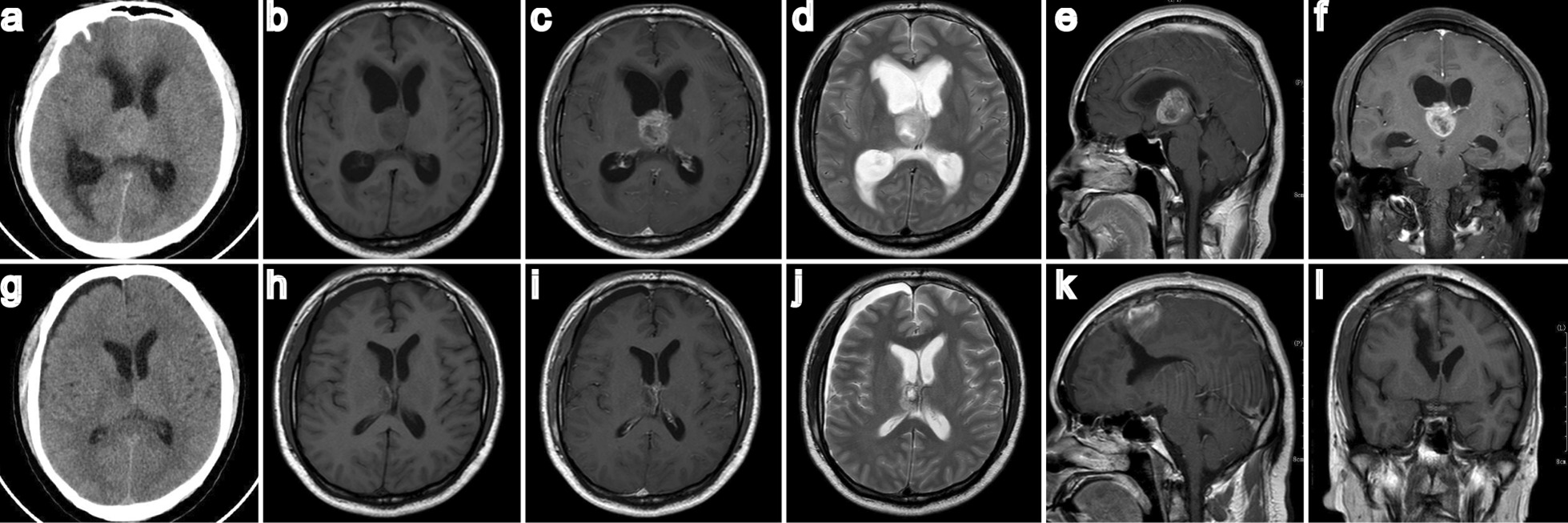
Case 1. Cranial CT and MRI revealed a unilateral thalamus located in the right thalamus. Preoperative CT and MRI (**a**–**f**). Postoperative CT and MRI confirmed total resection (**g**–**l**)

A 38-year-old male was admitted to the hospital with sudden headache accompanied by nausea and vomiting for 5 days. No obvious positive nervous system signs were found at admission. On admission, CT and MRI of the head revealed lesions in the right thalamus and third ventricle and subsequent hydrocephalus (Fig. 1a–f). Clinical diagnosis: Right thalamic glioma, obstructive hydrocephalus.

#### Surgical strategy and surgical approach (Fig. 2a–f)

**Fig. 2:**
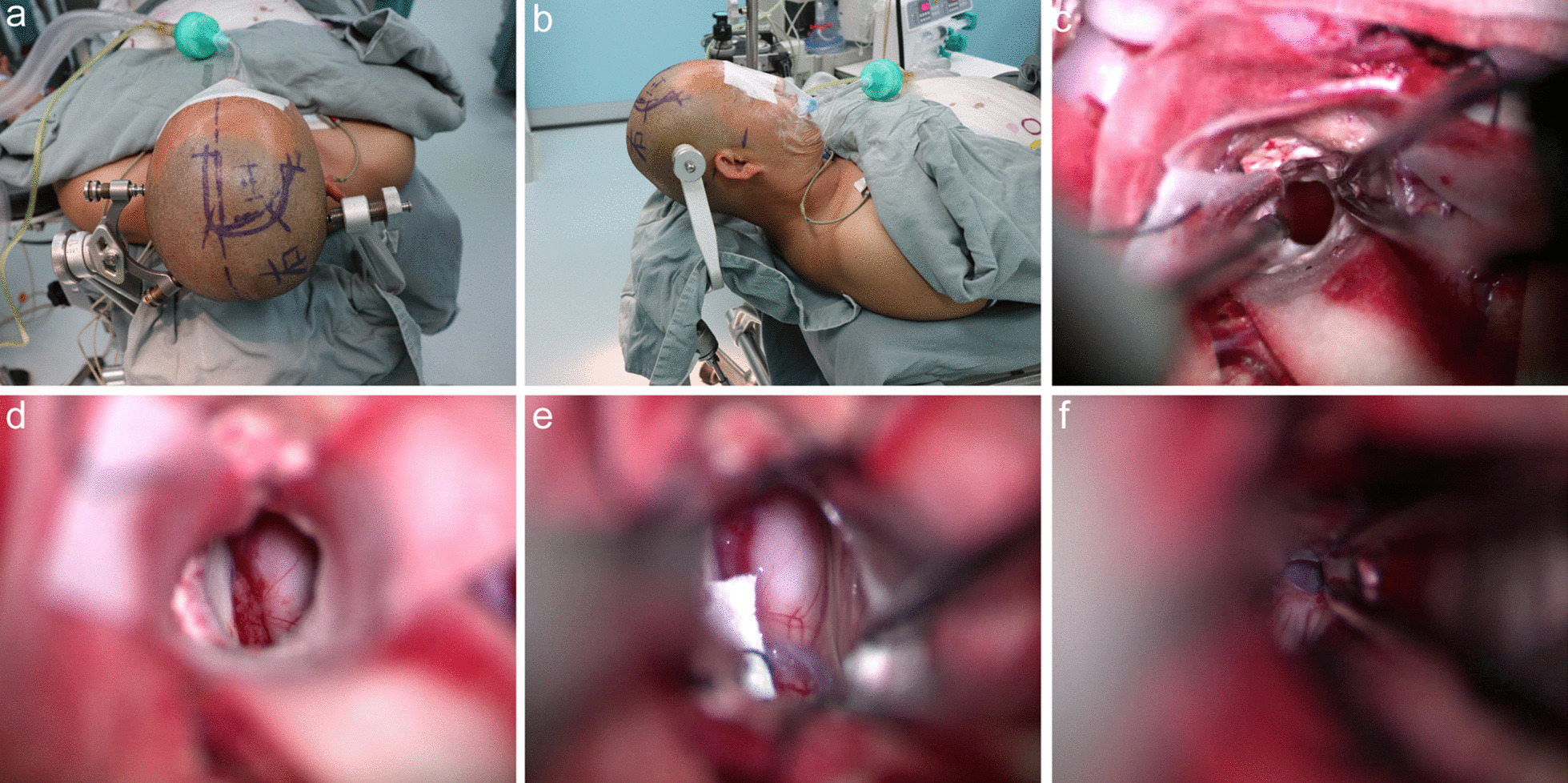
Case 1. a The skin incision; b the position of the head raised approximately 30 °C; c the location of the frontal cortex fistula: 2.5 cm in front of the coronal suture and 1 cm beside the midline; d the tumour, the choroid plexus and the body fornix were exposed; e the thalamostriate vein was exposed; f the location of the third ventriculostomy

According to the CT and MRI scans, the tumour originated from the right thalamus and grew medially and anteriorly, with the main body located in the body of the lateral ventricle. The anterior boundary of the tumour adhered to the interventricular foramen, the posterior boundary of the tumour closed the junction between the body of the lateral ventricle and the atrium and the medial part of the tumour wrapped around the midline structure. The patient also had obstructive hydrocephalus due to the tumour blocking the interventricular foramen. Therefore, the right trans-frontal lateral ventricle approach for tumour resection and third ventricle ventriculostomy were selected. First, the tumour was partially excised to separate and protect the thalamostriate vein and internal cerebral veins. After identifying all the structures, the tumour was completely removed along the approximate boundary of the tumour. Tumour resection followed the sequence of the dorsal wall, lateral wall, anterior pole, posterior pole, ventral wall and medial wall. A third ventriculostomy was performed by adjusting the microscope angle (Fig. 2f).

Postoperatively, there was no deterioration of the consciousness level, motor weakness or aphasia. Diabetes insipidus occurred during and after the operation and improved markedly at the time of discharge after 2 weeks. A postoperative MRI scan showed that the tumour was completely resected, and the ventricles were significantly smaller than they were in the preoperative MRI scan (Fig. 1g–l). The pathology was glioma blastoma. The patient received radiotherapy and chemotherapy treatment postoperatively.

After 7 months of follow-up, the patient's language and motor function were normal, and there was no recurrence.

### Case 2

**Fig. 3:**
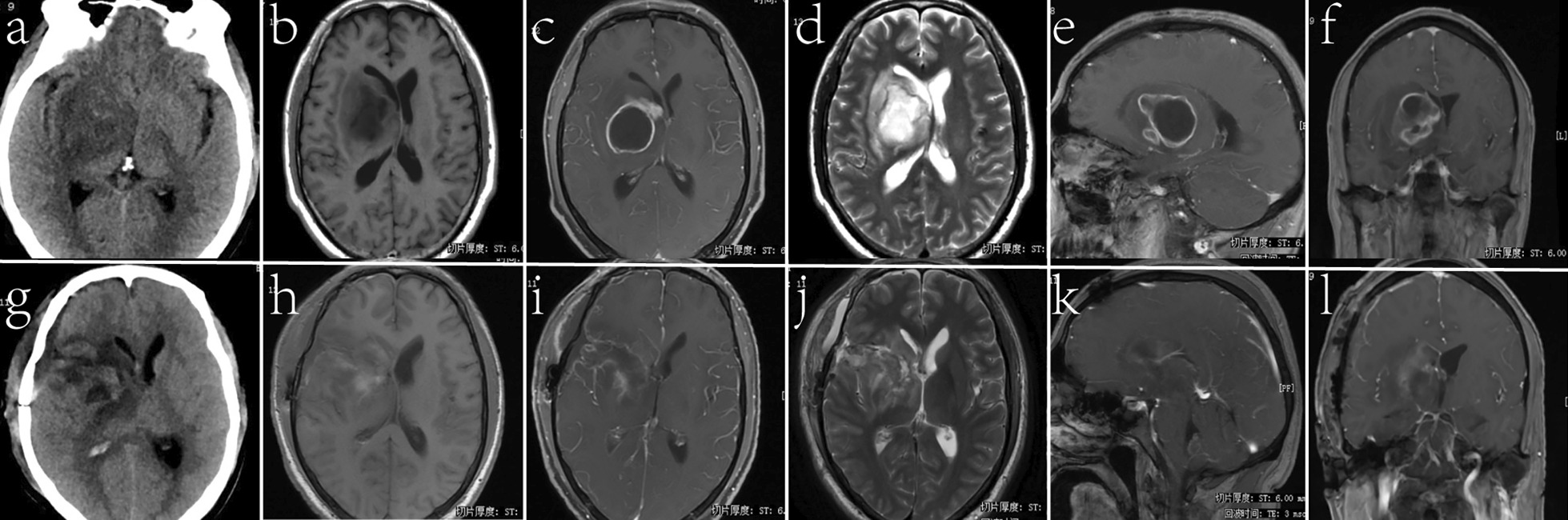
Case 2. Cranial CT and MRI revealed an abscess in the right basal ganglia. Preoperative CT and MRI (**a**–**f**). Postoperative CT and MRI confirmed total resection (**g**–**l**)

A 42-year-old male had fever and headache more than 1 month before admission, with the highest temperature being 39.5°. On admission, CT and MRI scans showed lesions in the right basal ganglia (Fig. 3a–f). Treatment with large doses of powerful antibiotics was ineffective. Consciousness disorder gradually appeared on admission, and the patient lapsed into a coma before the operation. Admission diagnosis: Right basal ganglia brain abscess.

#### Surgical approach and surgical strategy (Fig. 4a–f)

**Fig. 4:**
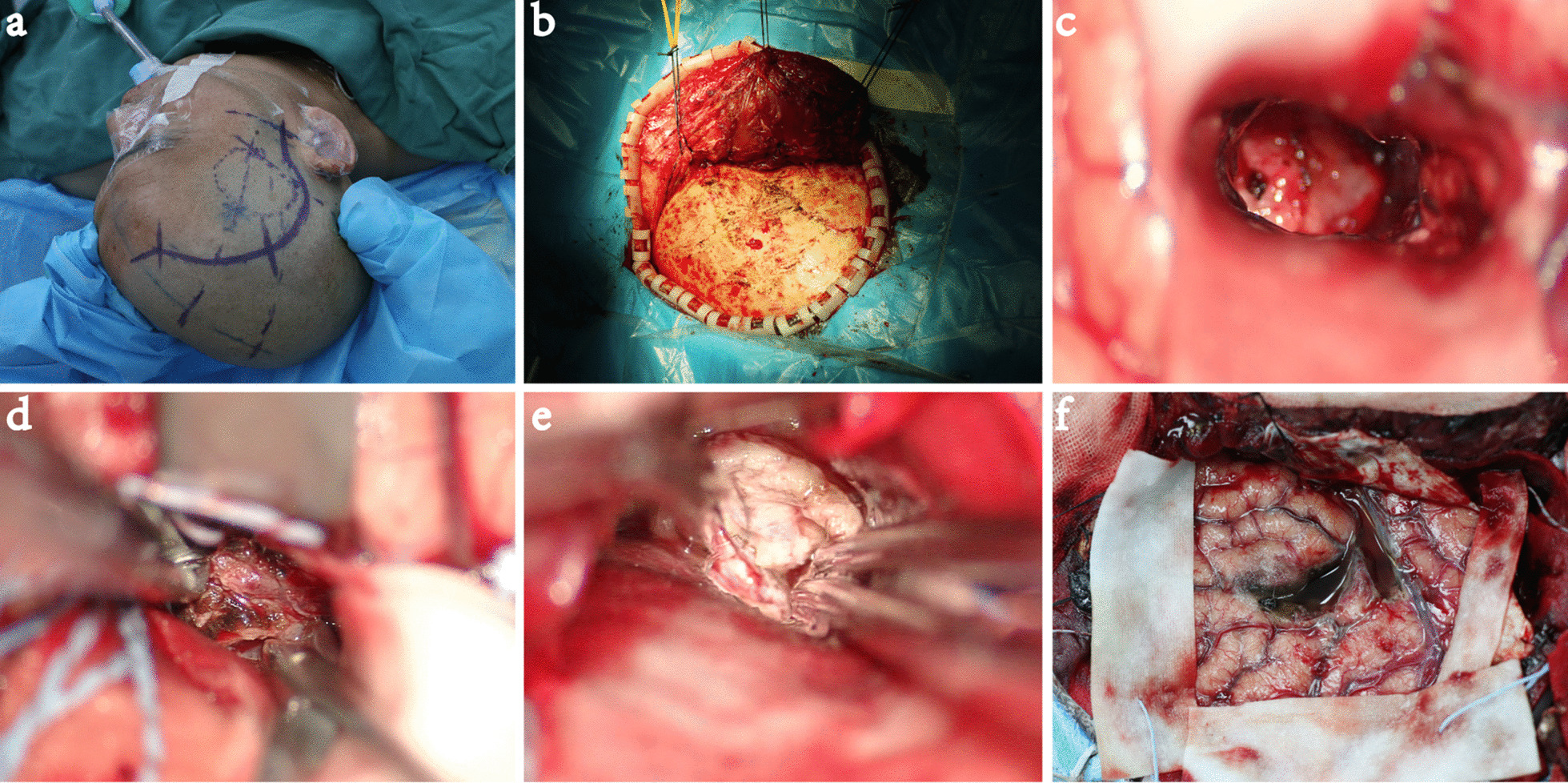
Case 2. a The skin incision; b the bony window was exposed, and the approximate location of the abscess was determined by the squamous suture; c the abscess; d part of the wall of the abscess was removed; e the ventral surface of the abscess was exposed; f the operative field was exposed after the operation

According to the CT and MRI scans, the main body of the lesion was located in the lenticular nucleus, with part of the lesion extending to the knee of the internal capsule close to the interventricular foramen. The edge was obviously strengthened, and the surrounding oedema was obvious. Combined with the patient's history of hyperpyrexia, it was considered to be an abscess. Due to the obvious lesion occupying effect and the ineffectiveness of a large dose of high-grade antibiotics, the patient's consciousness disorder was gradually aggravated. Therefore, resection of the abscess was performed.

#### Surgical approach

Right frontotemporal craniotomy via a transsylvian-transinsular approach for resection of brain abscess in basal ganglia (alternative: resection of brain abscess in basal ganglia with medial frontal gyrus fistula).

#### Surgical strategy

First, the lateral fissure was separated. Because of the high tension of the lateral fissure, it was difficult to expose the abscess. The medial frontal gyrus fistula approach was selected. The abscess wall was removed gradually along the periphery of the abscess and exposed the ventral side of the abscess. The ventral front of the tumour was adjacent to the internal capsule knee, the ventral rear of the tumour was adjacent to the thalamus, and the two parts needed to be carefully pulled to avoid injury. Due to the high tension of the lateral fissure and the difficulty of separation, the middle frontal gyrus cortical fistula was performed to remove the brain abscess in the basal ganglia area. The abscess resection followed the sequence of the lateral wall, the dorsal wall, the ventral wall, the posterior pole to the anterior pole and the medial wall.

#### Postoperative status

The patient still had fever after the operation, but the temperature was normal after 2 weeks of antibiotics. Body movement was normal. An MRI scan (Fig. 3g–l) 9 days after surgery showed complete abscess resection. Pathology: brain abscess. After 8 months of follow-up, the patient's language and motor function were normal.

### Case 3

**Fig. 5:**
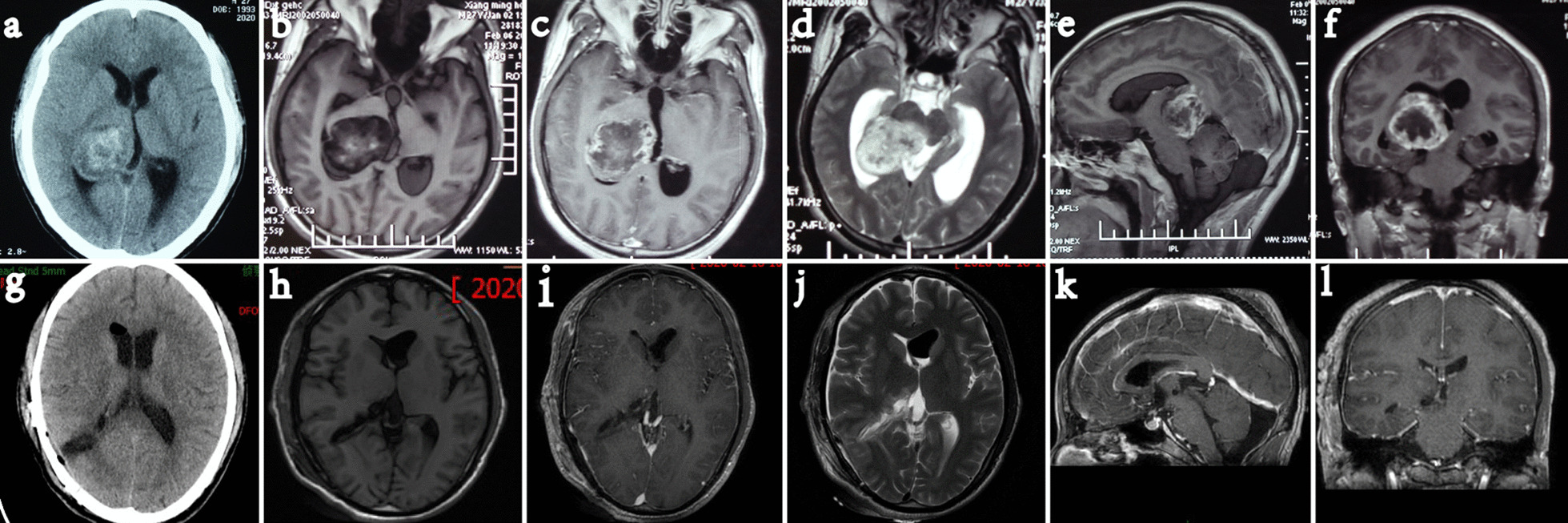
Case 3. Cranial CT and MRI revealed a unilateral thalamus located in the right thalamus. Preoperative CT and MRI (**a**–**f**). Postoperative CT and MRI confirmed total resection (**g**–**l**)

A 27-year-old male was admitted after 4 days of recurrent headache and dizziness. No obvious positive nervous system signs were found at admission. CT and MRI (Fig. 5a–f) scans showed a lesion in the right thalamus.

#### Surgical approach and surgical strategy (Fig. 6a–d)

**Fig. 6:**
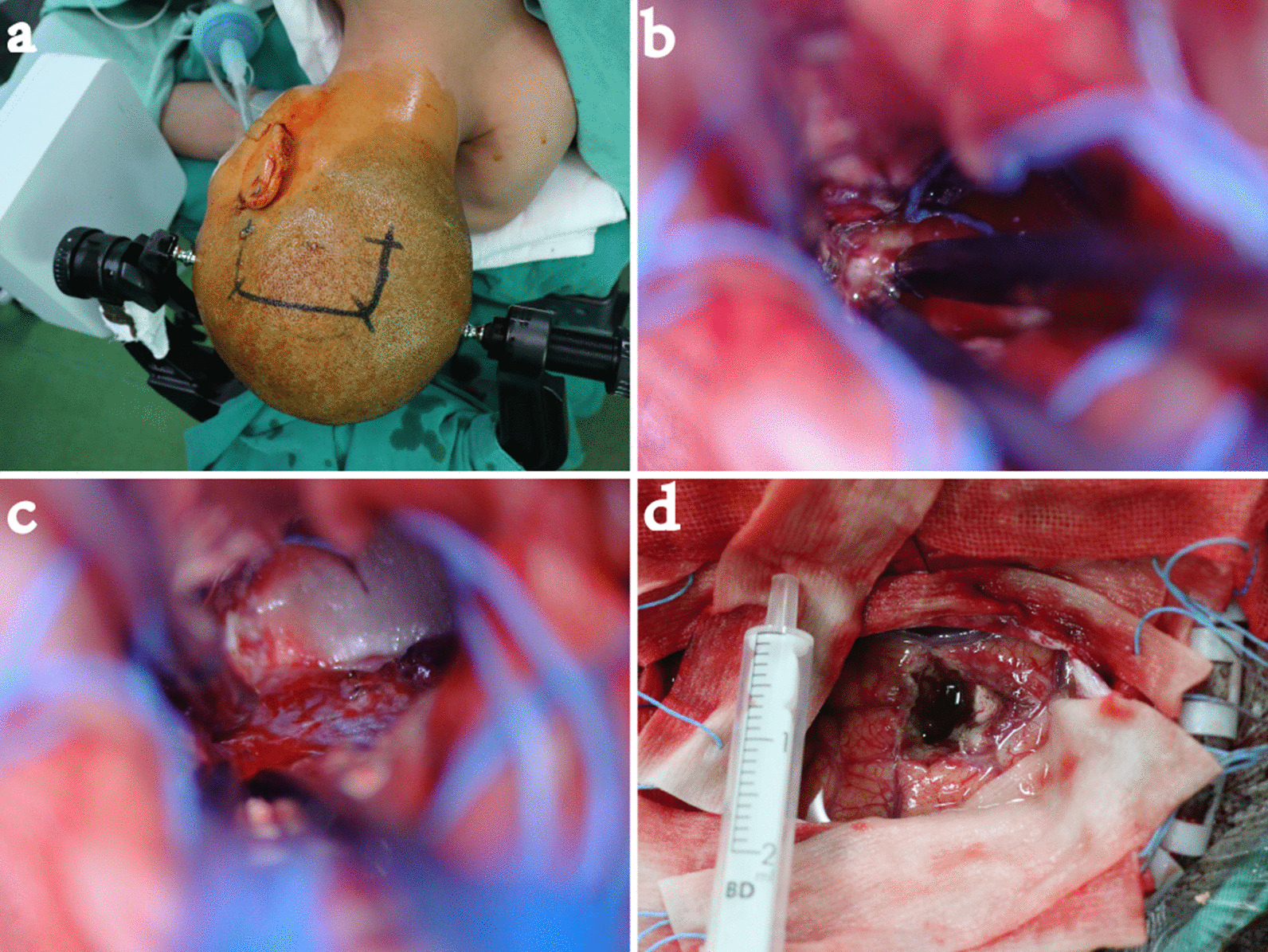
Case 3. a Skin incision; b anterior medial interface of the tumour; c lateral wall of the third ventricle; d the operative field exposed after the operation

According to the CT and MRI scans, the tumour originated from the right posterior thalamus and grew medially, anteriorly, upward and downward, with the main body located behind the thalamus in the right lateral ventricle triangle. Although the third ventricle was compressed, it was still unblocked, so the patient was not associated with obstructive hydrocephalus.

#### Surgical approach

Right temporal occipital craniotomy and right ventricular triangle approach were performed.

#### Surgical strategy

Through the temporal occipital junction area into the lateral ventricular triangle zone, the first part of the tumour was resected. During the process of resection, the three-dimensional structure of the tumour was determined, and the depth and position of the resection were determined according to the third ventricle and cerebellar tentorium when the tumour was close to the anterior medial interface and the lower interface. Tumour resection followed the sequence of the lateral wall, dorsal wall, ventral wall, posterior pole, anterior pole and medial wall.

#### Postoperative status (Fig. 5g–l)

The patient's body movement, language and reaction were normal after the operation. An MRI scan at 7 days after surgery showed complete resection of the tumour. No hydrocephalus was observed. Pathological findings: glioma blastoma, postoperative radiotherapy and chemotherapy treatment. The patient was not followed up after the surgery.

## Discussion and conclusions

**Fig. 7:**
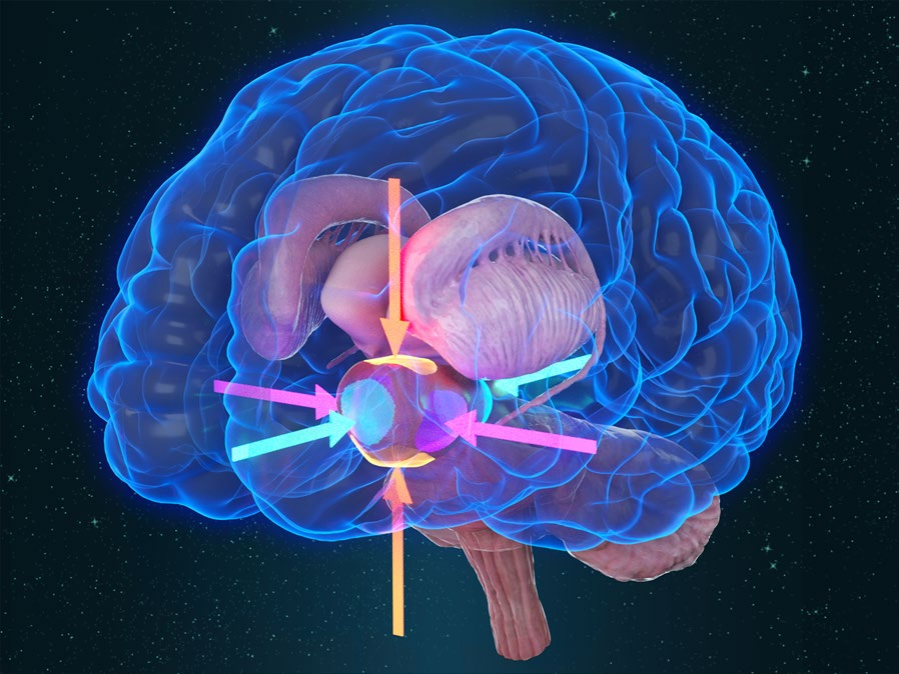
Schematic diagram of the "Four Walls, Two Poles". "Four Walls". Yellow arrows refer to the dorsal wall and the ventral wall; pink arrows refer to the lateral wall and the medial wall; green arrows refer to the anterior pole and the posterior pole

Thalamic glioma is located deep in the surrounding basal ganglia area with important nuclei and important deep cerebral veins. The basal ganglia region includes the caudate nucleus, lenticular nucleus, internal capsule and thalamus. In this study, two cases of thalamic glioma and one case of basal ganglia brain abscess were included to discuss the surgical techniques. Most thalamic gliomas and lesions in the basal ganglia area have irregular shapes, similar to the shape of an oval body. We subdivide this kind of oval body into "Four Walls, Two Poles". "Four Walls" refers to the dorsal wall, the ventral wall, the lateral wall and the medial wall. "Two Poles" refers to the anterior pole and the posterior pole (Fig. 7). David Kis [2] applied thalamic segmentation using probabilistic tractography and delineated the anatomical location of shifted thalamic nuclei to optimize their surgical approach and minimize surgical complications. In the same way as David Kis, we divided the lesions in the thalamus and basal ganglia into six parts. We generally followed the sequence of resection from the dorsal wall, the lateral wall, the anterior pole, the posterior pole to the ventral wall or the medial wall [3]. Depending on the location of the lesion, we adjusted the tumour resection sequence appropriately. During the process of the surgery, the three-dimensional concept of the “Four Walls, Two Poles” should be maintained.

The choice of surgical approach is a critical issue for tumours in the thalamus and basal ganglia regions. Zhang [4] published a large-scale thalamus glioma report. The clinical features, surgical approach, pathological features and prognosis of unilateral adult glioma were reported. The choice of surgical approach was based on the tumour location as well as the preferences of the surgeon. There are some surgical approaches for lesions in the thalamus and basal ganglia area. Amandeep Kumar [5] used the anterior interhemispheric transparaterminal gyrus approach to access the tumour successfully and achieved subtotal excision according to the relationship between thalamopeduncular gliomas and corticospinal tracts (CSTs). Qinglong [6] analysed the lateral or medial surgical approaches for thalamic glioma resection. Yukinori Akiyama [7] reported that rigid endoscopic resection using a thick sheath may be a viable method for the resection of thalamic gliomas. It is possible to carefully observe the tumour surface and avoid vessels during tumour resections under direct vision. Kentaro Chiba [8] reported a cohort of 10 paediatric patients with thalamic glioma who underwent surgical resection at our department. It has been reported [9] that patients with thalamic tumours secondary to hydrocephalus were first treated with neuroendoscopy for the third ventriculostomy and then underwent microscopic resection of the tumour. According to our experience, the frontal transcortical approach is a good choice for thalamic glioma with hydrocephalus [3]. The tumour can be removed, and third ventriculostomy can be performed during this approach. Two surgeries could be avoided. In case 1, the frontal transcortical approach was performed. The location of the cortical fistula is critical for both tumour resection and third ventriculostomy. The location of the frontal cortex fistula should be 2.5 cm in front of the coronal suture and 1 cm beside the midline. In this way, the location of the frontal cortex fistula is closer to the midline, which is beneficial to the operation angle of the third ventriculostomy. The fistula size could have a diameter of approximately 2 cm. The excision order of the lesion was the dorsal wall, the lateral wall, the anterior pole, the posterior pole, the ventral wall and the medial wall. The most dangerous area of this lesion was the medial and the ventral wall, which were closely adhered to the bilateral internal cerebral veins and the posterior medial choroidal artery. In case 2, the main abscess was located in the lenticular nucleus, and the superficial part of the abscess was relatively close to the insular lobe. Therefore, the expected approach before the operation was a transsylvian-transinsular approach [10]. However, it was found that the lateral fissure was difficult to separate, and there was almost no lateral fissure cistern. The abscess cannot be removed through the transsylvian-transinsular approach. Therefore, the separation of lateral fissures seems to be effective. When there is a deep brain lesion, it is difficult to separate and expose the lesion through the transsylvian-transinsular approach, so it is not practical to separate the lateral cistern. Therefore, cortical fistula seems to damage the cortex, but it is effective. Moreover, the M2, M3 and M4 branches of the middle cerebral artery of the lateral cistern were not affected, and the risk of cerebral vasospasm was avoided. In case 2, during the surgery, the excision sequence of lesions was the lateral wall, the dorsal wall, the anterior pole, the posterior pole, the ventral wall and the medial wall. The most dangerous areas of this lesion were the anterior and posterior parts of the medial wall, adjacent to the interventricular foramen and the dorsal thalamus, respectively. In case 3, the tumour was located in the ventricular triangle, and the temporal occipital approach was a good choice. The excision sequence of lesions was the lateral wall, the dorsal wall, the ventral wall, the posterior pole, the anterior pole and the medial wall. The most dangerous areas of this tumour were the anterior pole and the medial wall, which were adjacent to the dorsal thalamus and the third ventricle, respectively. In this case, after the resection of most of the tumour, hard cerebellar tentorium and thin lateral transparent membranous structure of the third ventricle were observed in the deep position. These two structures were obviously different from those of the tumour and thalamus, which could clearly indicate the depth of the operation.

In the process of excision of the thalamus and basal ganglia lesions according to the three-dimensional concept of "Four Walls, Two Poles", we should pay attention to the application of some surgical techniques. There are many techniques for using cerebral retractors wisely. Because the position of the thalamus and basal ganglia region is deep, the application of the cerebral retractor is required under normal circumstances. However, it is not the same as a normal open surgery. When the cerebral retractor is fixed, its position also limits the movement of the bipolar, the attractor, etc. Therefore, surgery of the thalamus and ganglion tumour could be performed without the cerebral retractor most of the time during surgery. The surgical corridor should be protected by cotton sliver along the operational channel, and the tumour should be excised under the condition of light retraction of the brain tissue with the cooperation of bipolar and suction apparatus. If retraction is required, the cerebral retractor can be placed on the opposite side of the main operating direction to reduce the restrictions on the other instruments. The aspirator is the most important tool in surgery. In our study, the tumour was removed mainly by suction [3]. The aspirator can be rotated quickly to generate a large negative pressure locally, thus improving the efficiency of tumour removal when the tumour is relatively large. After the majority of the tumour was removed, the normal structure was fully recognized, and everything was under control. Thalamic tumours are often associated with hydrocephalus. In case 1, the thalamic tumour simultaneously blocked the interventricular orifice. For thalamic glioma with hydrocephalus, the frontal approach can be selected as much as possible. The advantage is that the third ventriculostomy can be performed simultaneously. Therefore, the cortical orifice was located 2 cm before the coronal suture, 1 cm close to the midline. It is beneficial to the operation of the third ventriculostomy. Haemostasis is an important procedure for tumours involving the ventricular system. Bipolar should be used to meticulously stop the bleeding in the whole wound surface without gelatine sponge and haemostatic gauze to prevent cerebrospinal fluid circulation disorder. The technique of manipulation is another important factor affecting the prognosis of thalamus and basal ganglia tumours. The manipulation should be as gentle as possible.

The key to the success of the operation is the division of thalamic gliomas and the lesion in the basal ganglia area and an appropriate approach.

## Data Availability

The data used during the current study are available from the corresponding author on reasonable request. The additional supporting files of the images of the cases has been submitted.

## Abbreviations

Rt: Right
Lt: Left
RT: Radiotherapy
CT: Chemotherapy
T: Trans
Vent: Ventricular
Cort: Cortical
GTR: Gross total resection
STR: Subtotal resection
PR: Partial resection
OS: Overall survival
PITP: Precentral interhemispheric transcallosal interforniceal
AIPG: Anterior interhemispheric transparaterminal gyrus
Bi: Bilateral
CPeSS: Contralateral perimedian supracerebellar suprapineal
TLBG: Translateral brain gyrus approach
TCC: Transcorpus callosal approach
AT: Anterior type
IC: Thalamointernal-capsular
TP: Thalamopulvinar type
